# A Measure of Long-Term Hearing Aid Use Persistence Based on Battery Reordering Data

**DOI:** 10.1097/AUD.0000000000001032

**Published:** 2021-04-01

**Authors:** Oliver Zobay, Lauren K. Dillard, Graham Naylor, Gabrielle H. Saunders

**Affiliations:** 1 School of Medicine, University of Nottingham, Nottingham and Glasgow, United Kingdom; 2 VA Rehabilitation R&D, National Center for Rehabilitative Auditory Research, Portland, Oregon, USA; 3 University of Wisconsin-Madison, Madison, Wisconsin, USA; 4 Manchester Centre for Audiology and Deafness (ManCAD), University of Manchester, Manchester, United Kingdom.

**Keywords:** Audiology, Electronic health records, Hearing aids, Hearing aid use, Hearing healthcare, Hearing aid outcome, Persistence, Veterans Health Administration, Veterans Administration

## Abstract

**Objective:**

We describe the construction of a hearing aid long-term use persistence measure based on battery reorder data. The measure is derived from the notion that hearing aid users keep using their devices for some time after placing a battery order.

**Design:**

A hearing aid user is defined as persistent at time *T* if they placed a battery order within a time span *W* preceding *T*. We characterize and validate this measure using electronic health records from a large sample of US Veterans.

**Results:**

We describe how to choose parameters *T* and *W* for calculating persistence rates in the patient sample. For validation, the associations of persistence with: (1) the duration over which users received outpatient hearing aid care; (2) self-reported hearing aid use shortly after fitting; and (3) patient age and hearing loss are investigated. In all cases, plausible dependencies are observed.

**Conclusions:**

We conclude that our persistence measure is viable and hope this will motivate its use in similar studies.

## INTRODUCTION

Data on long-term hearing aid (HA) use that are suitable for audiological research are difficult to obtain but have a considerable value for understanding behaviors. An opportunity for obtaining such information has recently arisen in the context of a research project that analyses electronic health records (EHRs) of over 700,000 US Veterans who received HAs from the Veterans Health Administration (VHA). The VHA offers free battery refills for VHA-dispensed HAs, and the dataset includes time-stamped records of all battery orders placed within a period of 4 years and 9 months. Under the assumption that Veterans obtain replacement batteries predominantly through the VHA, their battery-order history reflects their HA usage, in that persistent users will continue to reorder batteries over time, whereas orders by nonpersistent users will cease once the use of HAs stops. However, as batteries are ordered infrequently and at irregular intervals (Saunders et al. 2020), the construction of a reliable HA use persistence measure is not straightforward.

This brief report describes a persistence measure that we developed to examine long-term HA use based on battery-order data, the steps undertaken to demonstrate its validity, and some considerations regarding its robustness. An understanding of the properties and limitations of this measure, which parallels methods widely used in pharmacological research to describe medication adherence (Cramer et al. 2008; Saunders et al. 2020), should be of interest to readers considering a similar approach.

## MATERIALS AND METHODS

### Dataset

Our dataset contains electronic health records from 731,213 US Veterans for whom HAs were ordered within VHA audiology between April 2012 and October 2014. Data used in this report include standard demographic information, battery-order dates between April 2012 and December 2017, audiometric data for 570,295 patients, and International Outcome Inventory for HAs (IOI-HA) responses completed 14–180 days after HA fitting available for 146,699 patients. Additionally, procedural and diagnostic codes were used to identify HA-related appointments. See Dillard et al. (2020) and Saunders et al. (2020) for a full description of the provenance and processing of the dataset.

### Battery Orders

All patients eligible for VHA HAs can order batteries from VHA free of cost and as needed, within reasonable limits. Each order is calibrated to last 6 months assuming full-time HA use of the specific HA type and fitting (unilateral versus bilateral).

### HA Use Persistence

To relate battery-order data to HA usage, we assume that a patient who ordered a battery refill will continue to use their HAs (i.e., be a persistent user) for a duration of time *W* thereafter. A patient is therefore considered persistent at time T>0 relative to the date of HA fitting if at least one battery order was placed between T−W and *T*.

### Persistence Rate

The persistence rate at time *T* after fitting is defined as the proportion of persistent users at *T* among those who are (1) alive at *T* and (2) for whom *T* occurs no later than December 31, 2017.

## RESULTS: SENSITIVITY OF PERSISTENCE MEASURE TO PARAMETER VARIATIONS

Our persistence measure depends on two defining parameters, that is, the time point *T* at which it is computed and on the width *W* of the time window for observing battery orders, and the stability of the measure with regard to variations of these parameters must be understood.

**Fig. 1.**
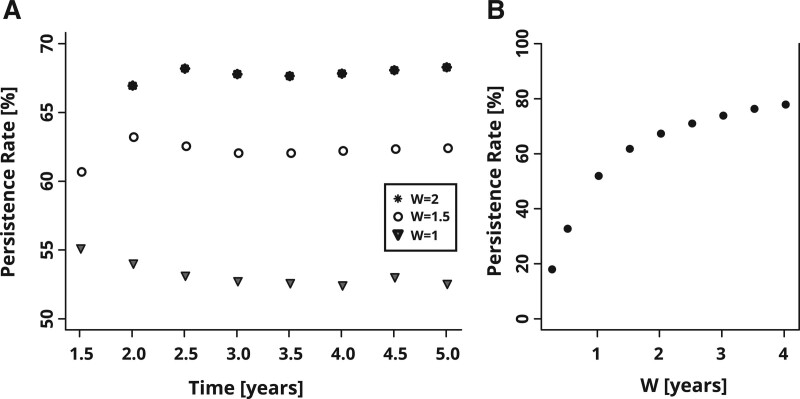
Dependence of persistence measure on defining parameters *T* and *W*: calculated persistence rates as a function of (A) time *T* after fitting for various values of the time-window length *W* and (B) *W* for *T* = 4 years.

For our dataset, the persistence measure is only meaningful for T≥W [for T<W, every patient has a battery order within *W* because the fitting date is defined as being equal to the date of the first battery order after HA order (Saunders et al. 2020)]. Figure 1A indicates that after *T* = 2 years, the persistence rate stays almost constant as a function of *T*, that is, the proportion of persistent HA users remains the same over time. Therefore, analyses will not be affected by the choice of *T* if T≥2 years. However, over time the sample size is reduced, thus we use T=2 years as a default value.

The dependence on *W* is necessarily more pronounced, since for W→0, the time window for observing battery orders shrinks, so the persistence measure must approach zero. Figure 1A shows that the calculated persistence rate indeed varies with *W*. For consistency, it is thus important to keep *W* fixed across all analyses within a project.

For our analyses we use W=18 months because this value falls at the cross-over from a fast to a slow increase in the persistence rate (Fig. 1B). This value of *W* implies that patients may remain classified as “persistent” if they use their HAs for at least 1/3 of the time (a nominal 6-month supply lasting 18 months).

Thus, in the analyses presented below, we choose T=2 years and W=18 months.

## RESULTS: VALIDITY OF PERSISTENCE MEASURE

### Connection Between HA Use Persistence and HA Appointments

**Fig. 2.**
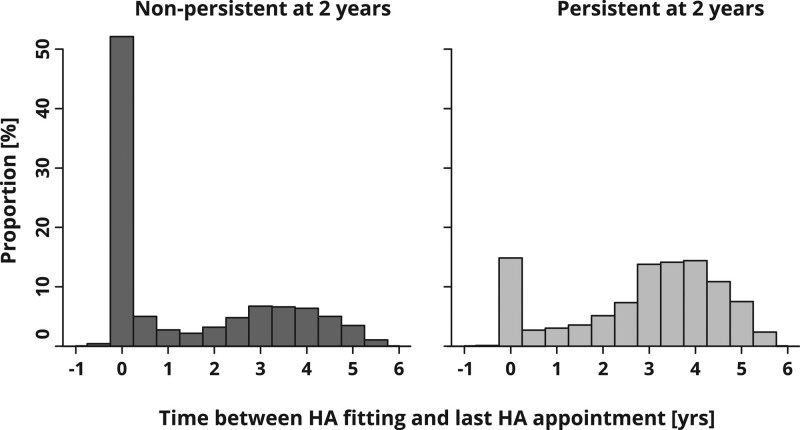
Distribution of time intervals between HA fitting and last recorded HA appointment for patients nonpersistent and persistent at 2 years after HA fitting. Negative time intervals occur if the last HA appointment found in the outpatient records took place before the first battery order after HA order (which is used as proxy for HA fitting) and thus are artifacts. HA, hearing aid.

It is plausible that there will be an association between HA use persistence and the time span over which HA appointments occur after fitting. This is corroborated by Figure 2, which shows a clear difference between nonpersistent and persistent users in the distribution of time intervals between HA fitting and the last recorded HA appointment. In fact, for 32.3% of nonpersistent users, their last appointment coincides with the HA fitting.

### Connection Between HA Use Persistence and Short-Term HA Usage (IOI-HA Question 1)

**Fig. 3.**
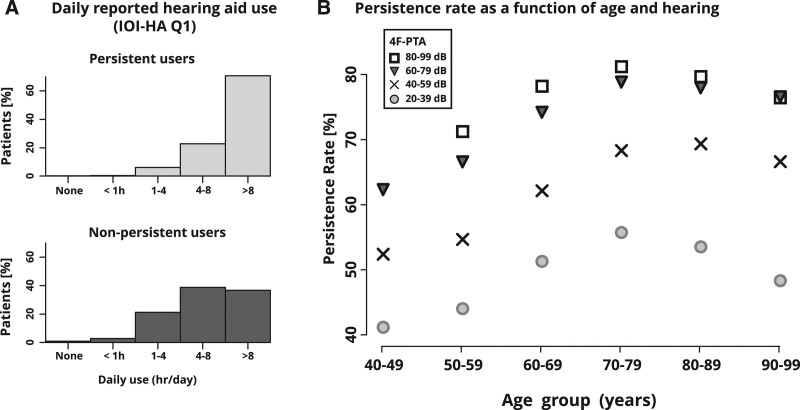
Relationship between HA persistence and short-term HA usage as well as patient age and hearing. A, Self-reported short-term HA usage (IOI-HA question 1) for patients persistent and nonpersistent, respectively, at 2 years after HA fitting. B, Persistence rate as function of patient age and PTA. Data point for age group = 40–49 years; 4F-PTA = 80–99 dB omitted due to insufficient sample size. 4F-PTA, four-frequency pure-tone average; HA, hearing aid.

Figure 3A shows the daily reported HA use from the IOI-HA for persistent and nonpersistent patient groups, respectively. It is evident that persistent users report greater daily HA use early on after fitting. However, this should not be interpreted to suggest that more HA use shortly after fitting results in greater long-term persistence, because the consequence of low daily HA use is low battery usage and thus a higher likelihood of being misclassified as nonpersistent by our measure. A better interpretation of Figure 3A might be that daily HA use tends to remain consistent over time. Regardless, the strong association supports the validity of our persistence measure. The patterns seen in Figure 3A remain similar when plotted for subsamples according to the time of IOI-HA report relative to fitting, that is, they do not appear to depend on when the IOI-HA was submitted.

### Dependence of Persistence on Age and Pure-Tone Average

Figure 3B illustrates that persistence is dependent on age and four-frequency pure-tone average (4F-PTA; bilateral mean of thresholds at 0.5, 1.0, 2.0, and 4.0 kHz) such that persistence increases with hearing loss (up to around 80 dB HL) and is highest for individuals aged 70–80 years. The former finding is consistent with the explanation that HAs offer increasing benefit up to a certain level of hearing loss (i.e., 80 dB). The latter likely reflects decreasing stigma as age increases, which, however, is counterbalanced by decline in cognitive and physical function beyond about 75 years. Persistence then declines most rapidly among those with milder hearing loss. These results are in line with other work (Gopinath et al. 2011; Bainbridge & Ramachandran 2014), thus providing further evidence for the validity of the persistence measure.

## DISCUSSION

The rationale underlying our HA persistence measure is that HA use can be inferred from battery-order history and, more specifically, that patients maintain HA use for a certain time period after a battery order. We provide evidence for the validity of the measure through its association with HA outpatient appointments, HA short-term usage (via IOI-HA) and patient age and PTA. The measure is robust regarding variations of *T*, but the more pronounced dependence on *W* implies that the present approach provides a plausible range rather than a definitive “true” persistence value. Nevertheless, it is suitable for making comparisons across experimental contrasts.

Of course, our measure has some limitations. First, it is binary and does not account for extent of daily use, as demonstrated by the fact that patients who order batteries every 6 months are treated the same as patients who order every 18 months. More generally, the assumption of continued HA use over a period *W* after battery order is a simplification of the actual patient behavior that was introduced to derive a tractable measure. Second, it is a long-term measure that, in our dataset, cannot be used to investigate the change in HA use during the initial 18 months after HA fitting. Furthermore, since battery orders in the VHA are infrequent and irregular, they are insensitive to some behaviors (e.g., 39% of users classified as nonpersistent at *T* = 2 years ordered batteries at a later date). Batteries obtained from sources outside VHA would lead to an underestimation of persistence. However, 99.8% of patients had at least one recorded battery order (Saunders et al. 2020) which implies that almost all patients order batteries through VHA. Persistence would also be underestimated if patients obtained a cochlear implant. However, few patients have procedural codes related to CIs (<0.2%), thus we consider this bias to be minor. Finally, rechargeable HAs are increasingly common, so battery-order data will become less available as time goes on. In this case, our data suggest that a count of postfitting HA appointments might be an acceptable substitute. Furthermore, cloud-based data logging is becoming widespread, and might be a new way to track usage accurately.

A persistence measure could have been constructed in many ways. Key reasons for our choice were its simplicity and its similarity to measures frequently used in pharmacological research. For comparisons of the absolute rate of HA persistence found by our method against rates of medication persistence in other conditions, see Saunders et al. (2020).

In summary, we have presented a measure of long-term HA use persistence that forms a cornerstone of the analysis of our VHA dataset, and have provided evidence of its validity. We hope that this report will stimulate the use of this or similar research tools by others. By providing a deeper understanding of which factors affect HA persistence, such research might eventually improve clinical practice and service.

## ACKNOWLEDGMENTS

The authors thank Kevin Quitmeyer, ShienPei Silverman, Kelly Reavis, Erin Robling, and M. Patrick Feeney for their support throughout the study.
